# Parametric Design and Prototyping of a Low-Power Planar Biped Robot

**DOI:** 10.3390/biomimetics8040346

**Published:** 2023-08-05

**Authors:** Koray K. Şafak, Turgut Batuhan Baturalp, Selim Bozkurt

**Affiliations:** 1 Department of Mechanical Engineering, Yeditepe University, Ataşehir, 34755 İstanbul, Türkiye; 2 Department of Mechanical Engineering, Texas Tech University, P.O. Box 41021, Lubbock, TX 79409, USA; b.baturalp@ttu.edu; 3 School of Engineering, Ulster University—Belfast, United Kingdom of Great Britain and Northern Ireland, York Street, Belfast BT15 1AP, UK

**Keywords:** biped robot, biomimetic robot, zero-moment point (ZMP) criterion, force-sensitive resistor (FSR) sensor

## Abstract

This study proposes a design approach and the development of a low-power planar biped robot named YU-Bibot. The kinematic structure of the robot consists of six independently driven axes, and it weighs approximately 20 kg. Based on biomimetics, the robot dimensions were selected as the average anthropomorphic dimensions of the human lower extremities. The optimization of the mechanical design and actuator selection of the robot was based on the results of parametric simulations. The natural human walking gait was mimicked as a walking pattern in these simulations. As a result of the optimization, a low power-to-weight ratio of 30 W/kg was obtained. The drive system of the robot joints consists of servo-controlled brushless DC motors with reduction gears and additional bevel gears at the knee and ankle joints. The robot features spring-supported knee and ankle joints that counteract the robot’s weight and compensate for the backlash present in these joints. The robot is constrained to move only in the sagittal plane by using a lateral support structure. The robot’s feet are equipped with low-cost, force-sensitive resistor (FSR)-type sensors for monitoring ground contact and zero-moment point (ZMP) criterion. The experimental results indicate that the proposed robot mechanism can follow the posture commands accurately and demonstrate locomotion at moderate stability. The proposed parametric natural gait simulation-based design approach and the resulting biped robot design with a low power/weight ratio are the main contributions of this study.

## 1. Introduction

Walking is a characteristic behavior that is present in most of the animal species on Earth. Studies on mimicking bipedal walking generally serve two main purposes. The first is to support the development of joint implants and assistive devices for walking or the development of physiotherapy methods using gait analysis. The second is to make widespread use of assistive robots in human environments aligned with technological advancements in actuators, control and computational systems, and autonomous behavioral algorithms. Research conducted over the past 4–5 decades has been dedicated to a number of design approaches, including anthropomorphic designs [1,2], vertical hoppers [3], passive walkers [4], and planar walking machines [5]. All these fields of research have made a significant impact in the field of biped locomotion. While WABOT-1 [1] can be considered as the first complete humanoid robot, ASIMO [2] was one of the most sophisticated humanoid robots of its time during the 2000s. Currently, the Atlas robot [6] is considered to be among the humanoids with the most advanced features that are aimed at military or emergency relief operations. The complexity of humanoid robots compelled designers to challenge the difficulties in mechanical design, hardware integration, and stability. Design concepts for the construction and hardware specifications of five different humanoid robots are discussed, and, additionally, four different biped locomotion control strategies are also proposed [7]. Cost-efficient designs are described that use a similar control architecture employing the CAN bus, which allows either a cooperative or standalone control method. Furthermore, this distributed method enables the integration of simpler control units such as sensing, processing, and actuating [8]. Different hardware designs and gait generation techniques have also been aimed at the development of new technology such as Stepper-3D [9]. A parallel double-crank mechanism for the leg structure and a gait generation method inspired by passive dynamic walking is adopted in this study. The LOLA [10] project is an instance of lightweight design, in which issues like fast, human-like walking are also targeted. Another study with a focus on lightweight design and low power consumption biped using 3D printed parts is presented in [11]. Topology optimization is another important approach that is being utilized in the structural design of robotic legs [12] and in hybrid legged-wheeled robots [13]. The design concept of lightweight biped robots based on biomimetic and human-like features such as compliant joints is also studied in [14]. In that study, the system is designed to have the ability to adapt to uneven ground. Lightweight design optimization based on walking simulation of biped robots considering the model of actuators and drivetrain components is also presented in [15].

The development of humanoid or human-like robots and prostheses takes advantage of biomimetics in terms of design, control, and energy efficiency. The KAIST Humanoid Robot Platform KHR-3 (HUBO) has been designed with human-like features. The design concept, actuator selection, upper and lower body designs, walking trajectory generation, and control algorithm for HUBO are described in [16,17]. The design of humanoid robots, prostheses, and orthoses for assistive walking can also utilize energy harvesting and energy regeneration capabilities [18].

Robot joints are actuated by brushless DC motors that are driven by individual motor controllers. These motor controllers handle positioning control tasks in which a centralized PC-based controller manages coordination and high-level control tasks. The centralized controller sends commands via USB to one of the motor controllers, whereas the motor controllers communicate via the CAN network. Robots can demonstrate various static postures as well as locomotion behaviors. A walking algorithm based on the offline trajectory generation method proposed in [19] is implemented. The developed biped robot platform can serve as a test bed for future research.

This paper outlines the design and prototyping stages, as well as the novel aspects of the planar biped robot, YU-Bibot. The robot dimensions are based on human lower extremities with six actuated joints. The design and selection of actuators are based heavily on simulations that have been performed using human walking data (natural gait, obtained from a reference work by D. Winter [20]). A parametric design optimization method based on joint torques has been utilized, and structural parameters as well as joint actuators have been selected accordingly. As a result of the optimizations, 100 W brushless DC motors have been used in joint actuators. The total rated power consumption of the robot joints is 600 W, which leads to a low power-to-weight ratio of 30 W/kg. For a comparison of the power-to-weight ratio for various humanoid robots, readers may refer to [11].

## 2. Materials and Methods

**Figure 1:**
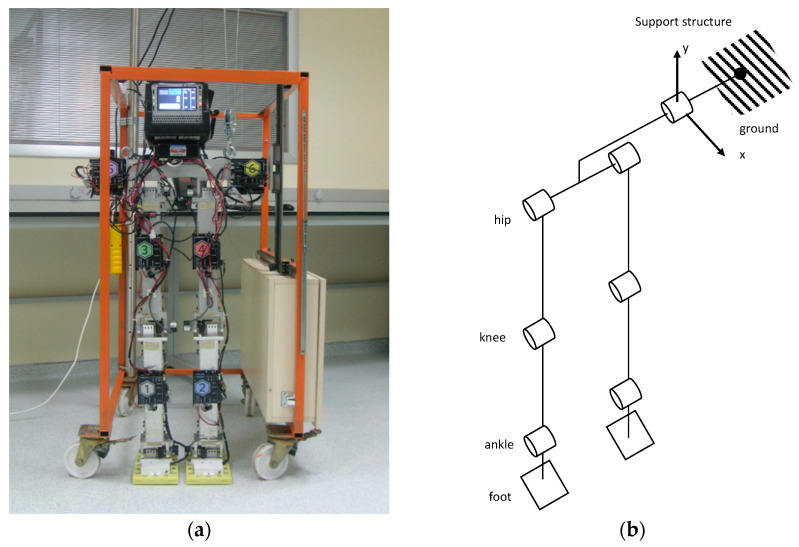
(**a**) Robot prototype with the support structure; (**b**) Schematic joint structure.

The planar biped robot is designed according to the anthropometric dimensions of human lower extremities. The total width and height of the robot are 600 and 1082 mm, respectively. Segment lengths are initially based on human proportions, as listed in [20]. However, the length of the waist is kept long on purpose to protect the hip motors. All the joints (hip, knee, and ankle) consist of 1 rotational axis. The actual hardware and lateral support structure of the robot can be seen in Figure 1a, and a schematic joint structure of the robot is shown in Figure 1b.

Six motor-driven rotational joints and planar constraint with a hinge joint (lateral support structure) are shown in the joint structure layout. Since the biped robot is initially designed as a planar robot, a lateral supporting system is crucial to constrain the motions of the robot in the sagittal plane. The lateral support system consists of linear motion guides that are fixed to a wheeled carrier structure.

The lower extremity of an anthropomorphic robot must have limbs such as a shank, thigh, waist, foot, and torso. These limbs hold together the robot. For this purpose, components must not only have enough strength but also be lightweight. Several materials can satisfy these properties such as Delrin, aluminum, and carbon fiber. The Aluminum 6000 series sticks out with its ease of finding, low cost, good strength properties, and availability in various off-the-rack shapes. A rectangular-shaped profile is chosen due to its ease of assembly to joint mechanisms by gussets. In joint mechanisms, two types of materials are used based on their criticality. While steel is used for shafts and couplings, all other components of the joints are manufactured from 7075-T6 aluminum. This is mainly because of the manufacturability, low density, and high tensile strength properties of this material. The masses and dimensions of the primary robot segments are shown in Table 1.

**Figure 2:**
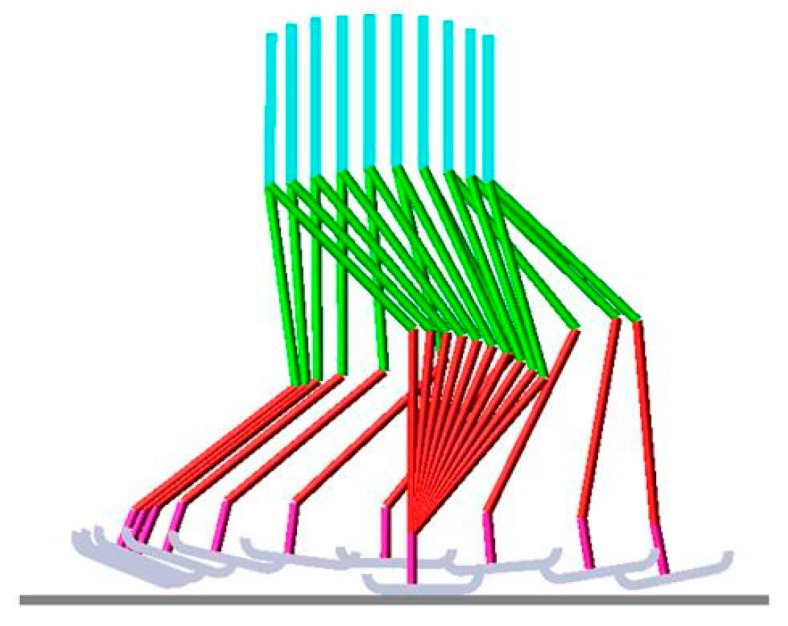
Superimposed view of the anthropomorphic model in ADAMS simulation for one step.

The selection of components for the robot is an important part of the design process. The ADAMS^®^ 2017 software is used to conduct parameterization and actuator selection studies. A parametric ADAMS model was built at human anthropomorphic dimensions [20] for 1.50, 1.75, and 2.00 m tall humans. Segment lengths are given as fractions of the total height. The parametric study provides the criteria for selecting the actuators with respect to torque requirement and power consumption in the simulation. The parametric ADAMS model (Figure 2) is used to calculate the required torque and power consumption values for six actuators (2 hips, 2 knees, 2 ankles) of the bipedal robot for 5 different walking speeds and 3 different heights. Actuators in the simulation are driven by the joint angular position values during the gait cycle. These angular position values are taken from a reference natural human gait which is obtained by using optical measurement techniques and motion analysis software [20]. Three cubic splines are used on the hip, knee, and ankle joints to form a gait cycle. Stance and swing leg movements are generated by using the same splines with a 50 percent phase difference, yielding identical gaits among left and right sides. To obtain a manipulator-like mechanism, the stance foot of the parametric ADAMS model is fixed to the ground (Figure 2). Power and torque values are calculated for each joint. But only stance leg joint values are considered due to their significantly higher values compared to the swing leg joint values.

**Figure 3:**
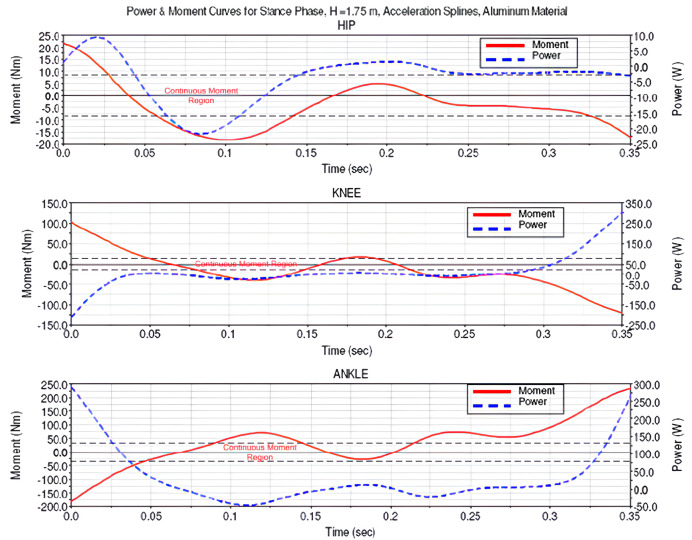
Torque and power diagrams based on anthropomorphic angular acceleration splines.

Figure 3 shows torque and power requirements in the joints for 1.75 m tall human proportion model. The horizontal dashed lines in the middle of the graphs refer to the continuous torque region of the selected motor and gearhead couple.

Moment and power consumption of the actuator continuous loading capacity requirements are summarized in Table 2. 

Samples 7 and 8 are simulations that were created with human angular acceleration values instead of human angular position values. A significant drop in the values by using angular acceleration values can be seen if Sample 8 is compared with Sample 5. With the help of parameterized simulations, Sample 7 is chosen as a guide to the selection of actuation components. Brushless servo motors of 100 W and aluminum structural materials are selected in the light of this study. The use of 100 W per joint provides a total electrical power of 600 W, and hence a power-to-weight ratio of 30 W/kg is obtained.

A comparison study for different humanoid robots is given in [11], where the power/weight ratio of known humanoid robots varies between 25 W/kg and 133 W/kg. Hence, the proposed biped robot design can be considered low power.

Joint actuators consist of three main components: brushless dc motors, gearheads, and bevel gears. There are several actuation choices in the literature for biped robots. Alternatives include the use of electric motors and hydraulic or pneumatic actuation. Electric motors are preferred due to flexibility in application, providing compact packages and considered clean systems. In addition, they have some disadvantages like poor force-to-weight ratio and running at high speeds. To obtain the required torque, speed reducers are used. The downsides of using speed reducers include increased complexity, weight, compliance, and backlash. The necessary torques to drive the biped robot are computed in the parametric study.

**Figure 4:**
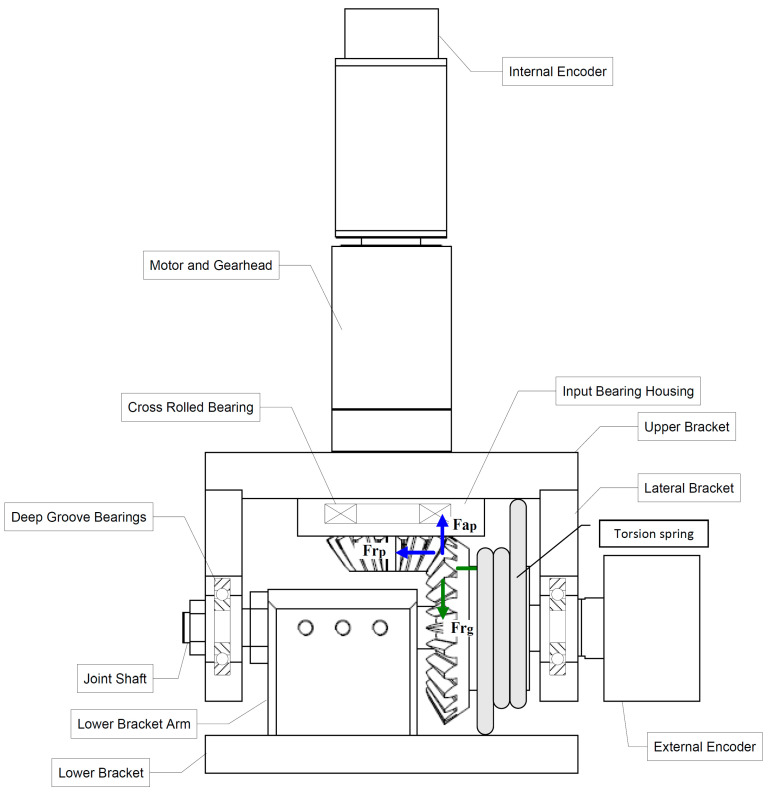
Knee joint design and components.

Selection criteria of the drive components selection can be listed as maximum continuous and intermittent torque, output speed of the gearbox, motor power, rated motor current, and peak current values that the motor control unit can supply. The preferred motor control unit is a smart brushless motor control unit that can provide a 5 A continuous and a 10 A intermittent current. Motor control units and all other electrical hardware components in the system are selected to work with a 15 V rechargeable battery supply when it is necessary. A schematic diagram of the knee and ankle joint design is shown in Figure 4. Bevel gear ratios of 1:1.5 and 1:2 are found suitable for knee and ankle joints, respectively. Motor gearheads on hip joints handle required moments; therefore, hip joints are designed as directly coupled to the motors.

Primary joint design considerations are described as being lightweight, strong, and compact. Some components such as motors have already been chosen before the joint design process. The knee joint is chosen as a starting point for the mechanical design of the robot. Based on optimizations and the number of revisions, a final knee joint design has been reached (see Figure 4).

After the knee joint is designed, the ankle joint is modified from it. The ankle joint requires higher torque than the knee design, so a larger reduction ratio of bevel gears (2:1) is used. The design of the hip joint is less complicated compared to knee and ankle joints. Mainly due to lower moment requirements, no reduction such as bevel gears is needed. Power transmission from the motor to the joint was performed by direct coupling. 

Torsion springs used in the knee and ankle joints provide an increase in joint stiffness and reduced backlash. Also, torsional springs carry part of the load exerted on the ankle and knee joints due to static weight. The robot joints are actively controlled by motion controllers, so additional joint stiffness simply helps controllers in reducing the total joint load. Hence, the joint actuators consume less electrical current in balancing the robot in the upright position.

**Figure 5:**
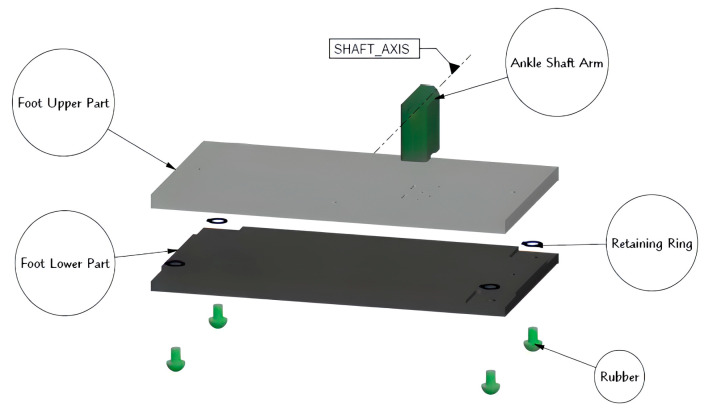
Exploded view of the foot assembly.

After force sensors are selected, the design of the foot takes place. Figure 5 shows the exploded view of the foot assembly. The main considerations in foot design are ease of manufacturing and features for FSR sensor mounting. For ease of machinability, the foot is divided into two main pieces. The upper part is designed to mount directly to the lower part of the ankle joint assembly. One of the considerations in foot design is to transmit ground reaction forces to the FSR sensor properly. To protect the sensor and reduce the effect of the impact, rubber is selected as a material to absorb the ground contact force. FSR sensors are mounted at the corners of the bottom surface of the upper piece by an adhesive. Retaining rings are used to keep rubbers on FSR sensors when the foot is in the air.

**Figure 6:**
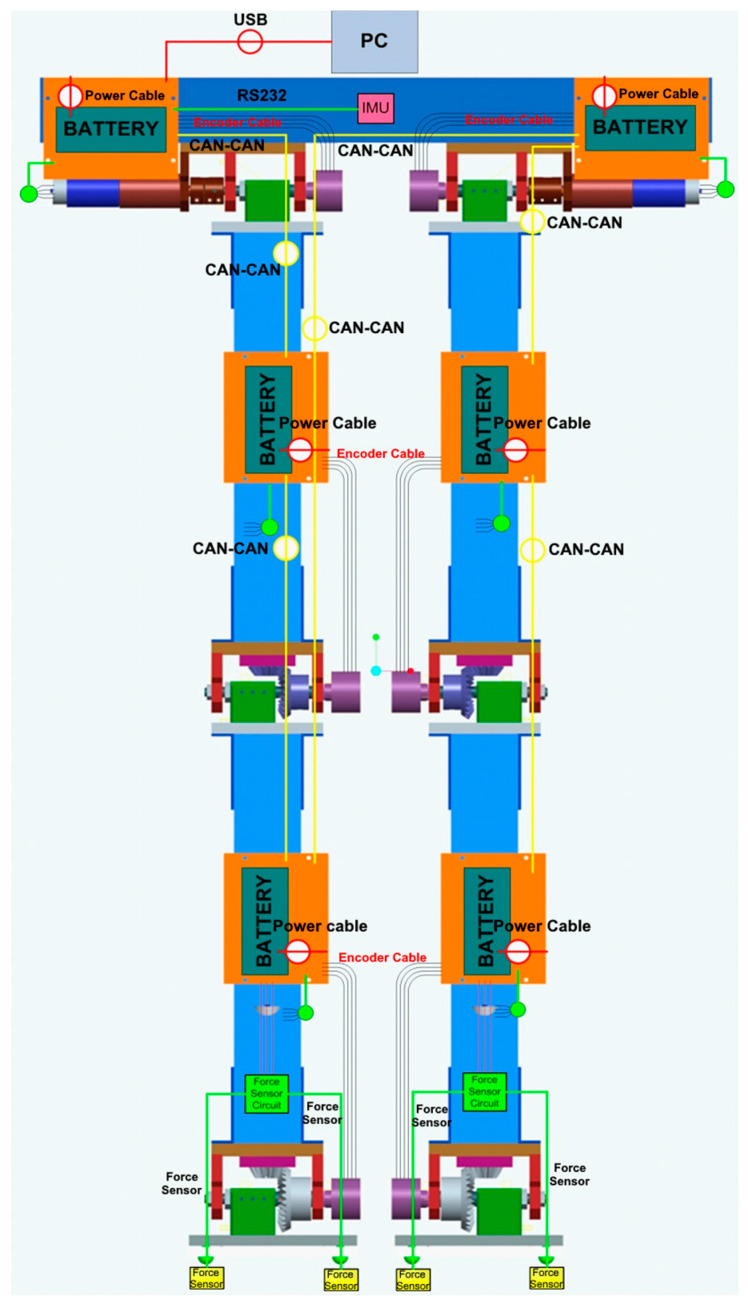
Electrical assembly diagram of the sub-systems of the robot.

Electrical and control system design section covers the design and selection of the sensors, implementation, interfacing, and general electrical design of the whole robot. The electrical assembly layout of the robot is shown in Figure 6.

Advanced features of motor controllers (Maxon EPOS2^®,^, Maxon, Sachseln, Switzerland) make it possible to use them as a data acquisition system. Therefore, not only do they control the motors, but they also gather data from external encoders, force-resistive sensors, and the inertial measurement unit. These motor controllers can create a data flow network by communicating with each other via the CAN (Controller Area Network) protocol. Hence, every controller has a role as a data concentration point on the network. This entire CAN bus network is connected to a main computer by a USB bus.

Inertial measurement unit (IMU) and external encoders provide vital information regarding the position and orientation of the biped robot. IMU can determine the robot’s position and orientation with respect to the ground, and external encoders mounted at one end of joint shafts determine the angular position of joint shafts reliably. Information provided by the encoders is not affected by backlash on gearheads and bevel gears. The motor controller uses internal encoders only to drive the motors. IMU consists of roll, pitch, and yaw gyro sensors and includes three-axis translational acceleration sensors. This sensor is temperature and magnetic field compensated. It has a sensitivity of ±0.005 g within a ±5 g range for translational movements and a sensitivity of ±0.2°/s within a ±300°/s range for rotational movements.

The zero-moment point (ZMP) concept was introduced in 1969 by Vukobratović and Boravac [21] and has since been widely used as a measure of the stability of biped robots. Although ZMP is generally defined in the x and y axes, a planar biped robot’s stability varies in the *x*-axis only. The equation for the ZMP stability margin along the walking direction, $X_{zmp},$ is constructed as follows:

(1) $$X_{zmp}=\frac{\sum_{i}^{n}m_{i}\left({\ddot{y}}_{i}+g\right)x_{i}-\sum_{i}^{n}m_{i}y_{i}{\ddot{x}}_{i}-\sum_{i}^{n}I_{iy}\alpha_{iy}}{\sum_{i}^{n}m_{i}\left({\ddot{y}}_{i}+g\right)},$$

where $m_{i}$ is the mass of the *i*th link, $I_{iy}$ is the inertia and $\alpha_{iy}$ is the angular accelerations of the *i*th link about the *y*-axis, $z_{i}$ and ${\ddot{z}}_{i}$ are the position and acceleration of the *i*th link in *z*-axis direction with respect to ground, and *g* is the gravitational acceleration. Here, the links are numbered as foot = 1, shank = 2, thigh = 3, upper body = 4.

Since dynamic stability is measured by the ZMP method, experimentally, it must be measured by force/torque sensors. Due to their good shock resistance, low price, and thickness, Tekscan FlexiForce brand force-sensitive resistors (FSR) are used in this project. The FSR sensor is a thin and flexible printed circuit which can be easily integrated into most applications. A 0–110 N ranged version is used with a linearity (error) of ±3%.

The design of a sensor circuit was essential because the sensor acts as a variable resistor in an electrical circuit. When the sensor is unloaded, its resistance is very high (greater than 5 MΩ); when a force is applied to the sensor, its resistance decreases. To convert this resistance value into a 0–5 V analog output, a basic op-amp printed circuit is designed. As seen in Equation (2), the test voltage, $V_{T}$ must be negative for this circuit. The negative $V_{T}$ results in a positive 0–5 V analog output signal that can be measured by motor controllers. Guidelines provided by FSR-type sensor manufacturer [22] were utilized in the design of the sensor conditioning circuit. The output voltage of the circuit is expressed as follows:

(2) $$V_{\mathrm{out}}=-\frac{R_{\mathrm{feedback}}}{R_{\mathrm{FSR}}}V_{T}.$$

The selection of $R_{\mathrm{feedback}}$ directly affects the relationship between the output voltage $V_{\mathrm{out}}$ and the force applied. As the value of $R_{\mathrm{feedback}}$ increases, the corresponding values of $V_{\mathrm{out}}$ also increase for the same applied force. Hence, a proper selection of $R_{\mathrm{feedback}}$ is needed that maps the force measurement range of the sensor to the output voltage range of the circuit linearly. MCP6002 low-power op-amp which handles 2 FSR sensors is used in this circuit. A more comprehensive description and design of the circuit can be found in [22].

Although biped robots have better mobility than conventional wheeled robots, their stability is also lower compared to that of other kinds. To be able to maintain stability in various environments, rough terrains, slopes, and regions containing obstacles, it is necessary that the robot be adaptable to the ground conditions by using suitable gait trajectories. The offline gait generation method is used in this study. This method tries to generate a dynamically stable walking pattern offline and it assumes that robot and the environment models are available. The created dynamically stable offline trajectories can be optimized due to jerk, stability, and power consumption. Most of the offline gait generation methods in the literature rely on the ZMP for pattern generation and control [2,19,23]. Those ZMP-based methods usually require precise knowledge of the robot’s dynamics (e.g., mass, center-of-mass location, and inertia of each link) to generate the walking patterns. Hence, they are dependent on the accuracy of the models.

**Figure 7:**
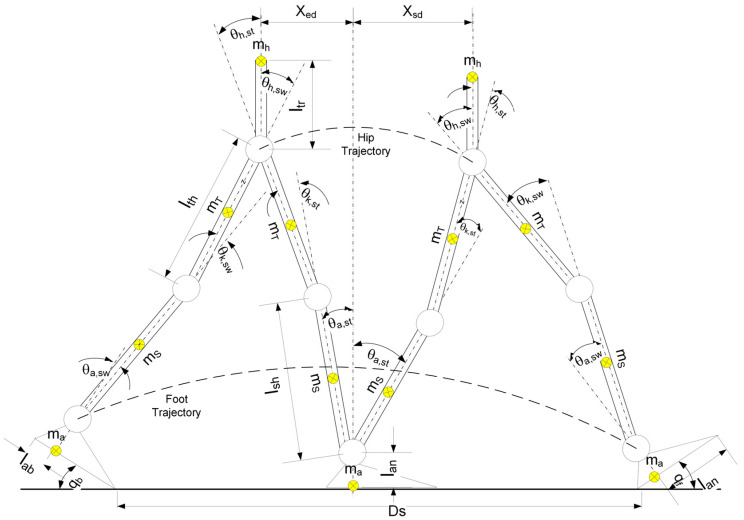
Model and gait parameters of the seven-link planar biped robot.

An offline gait trajectory is generated in the MATLAB^®^ software by using an approach given in the literature [19]. First, hip and ankle trajectories in Cartesian coordinates are generated with cubic splines by using a few characteristics via points. These characteristics via points and the initial phase of tracing the path for the generation of a simple walking trajectory are shown in Figure 7. The planar seven-link model is utilized with link offsets, locations of link centroids, and angular position values determining the system’s relation with the environment (Figure 7). In the figure, link lengths are labeled as $l_{an}$ (ankle to ground), $l_{ab}$ (ankle to heel), $l_{af}$ (ankle to toe), $l_{sh}$ (shank), $l_{th}$ (thigh), $l_{tr}$ (torso), joint angles are labeled as $\theta_{a}$ (ankle), $\theta_{k}$ (knee), $\theta_{h}$ (hip), and the subscripts st and sw indicate stance and swing legs, respectively. The distance along the *x*-axis from hip to the ankle of the support foot at the start end of the single support phase is denoted as $x_{sd}$ and $x_{ed}$, respectively. Segment masses are $m_{a}$, (foot), $m_{s}$ (shank), $m_{s}$ (thigh), $m_{h}$ (hip). The end of the double support phase configuration is represented on the left side of the figure. The right side of the figure represents the beginning of the double support phase. Projection of the foot and hip trajectory in the single support phase can also be seen in this figure.

A single walking step includes two phases. These phases are defined as the double support (DS) and single support (SS) phases. The double support phase occurs when both feet are in contact with the ground. The single support phase occurs when there is only one foot in contact with the ground. The beginning of a walking cycle can start from either the double or single support phase, but each phase must succeed the other.

**Figure 8:**
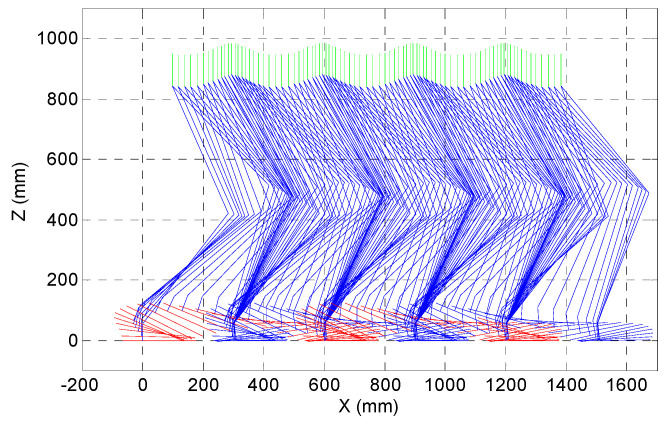
An example gait pattern generated for the seven-link biped robot.

A MATLAB program was used to create a cubic spline by the predetermined points from the characteristics of walking. One period of the walking cycle is shown in Figure 8. The walking pattern starts with double support, and initially, the right foot is the support foot until the next double support phase. After splines are generated for ankle and hip trajectories with respect to via points, Cartesian coordinates of the knee joint are found. The 2D stick diagram in Figure 8 is an output of the offline gait generation program.

**Figure 9:**
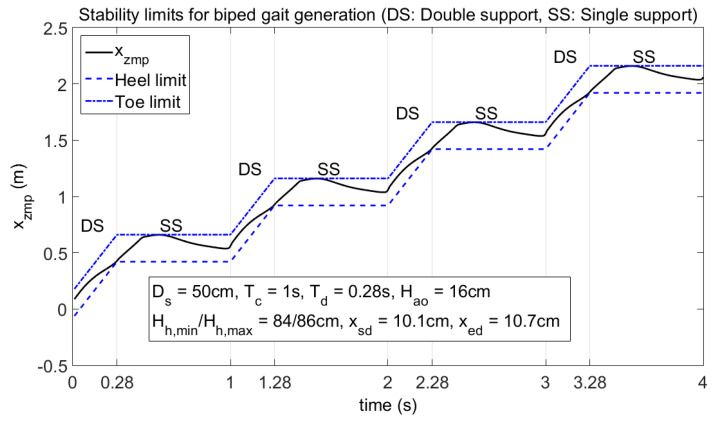
Stability limits for biped gait generation.

Offline-generated gait must be stable in terms of the ZMP criterion. The ZMP results obtained by simulations for a generated gait trajectory are shown in Figure 9. A stable walking gait for the biped robot can be generated as shown. Selected gait parameters are also indicated in the figure. Stability criterion *x_zmp_* stays within the support region defined by the heel and toe limits, as shown.

## 3. Results

**Figure 10:**
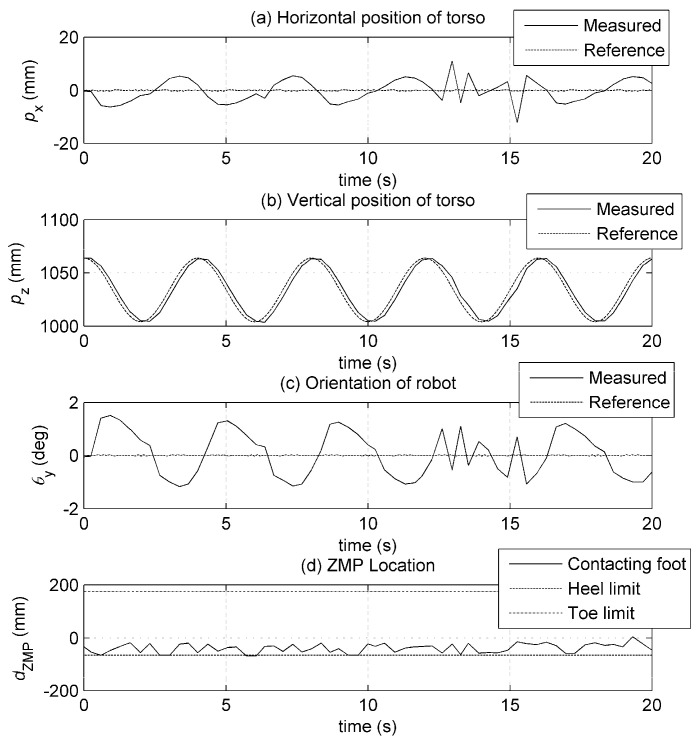
Results of height posture test. (**a**) Horizontal and (**b**) vertical torso positions, (**c**) orientation of the robot, (**d**) ZMP stability criterion.

A test on height posture was performed to assess the positioning performance of the joints and the static stability of the robot. The height of the torso is given a sinusoidal reference trajectory that is varied between 1004 mm and 1064 mm with an oscillation period of 4 s. The result of this test is shown in Figure 10. The horizontal torso position (Figure 10a) has a small variation with respect to its reference. The vertical torso position follows the reference well (Figure 10b). A fixed amount delay (≈200 ms) observed between the reference and measured values of height is primarily due to time lag in data acquisition (application of the reference signal and recording of the output). The angular orientation of the robot has a small variation of ±1° (Figure 10c). Stability criterion $d_{zmp}$ indicates that the robot can stand on its feet without much change in the location of the ZMP (Figure 10d). The location of the ZMP remains within the stable region defined by the heel and toe limits and has an average value of ≈107 mm. These results suggest that height reference tracking is achieved with good accuracy.

**Figure 11:**
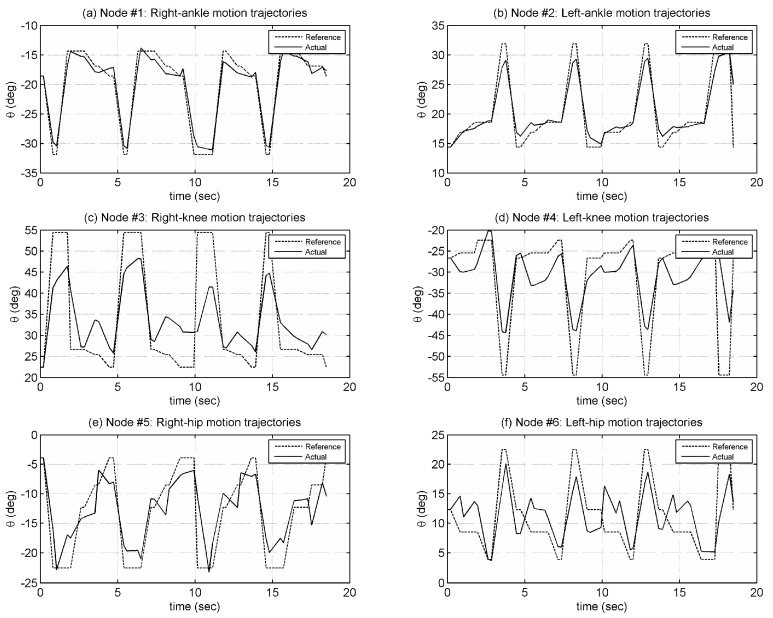
Joint motion trajectories (Nodes 1–6) during walking test.

Walking tests were performed on a smooth laboratory floor. The walking gait was generated with a step length of 40 cm and a step period of 2.7 s. The robot height was set at 90 cm. During these tests, the robot could bear its weight and achieve stable walking. Data were collected by the central controller, while the robot is in motion. Joint motion trajectories generated for the walking gait are shown in Figure 11.

**Figure 12:**
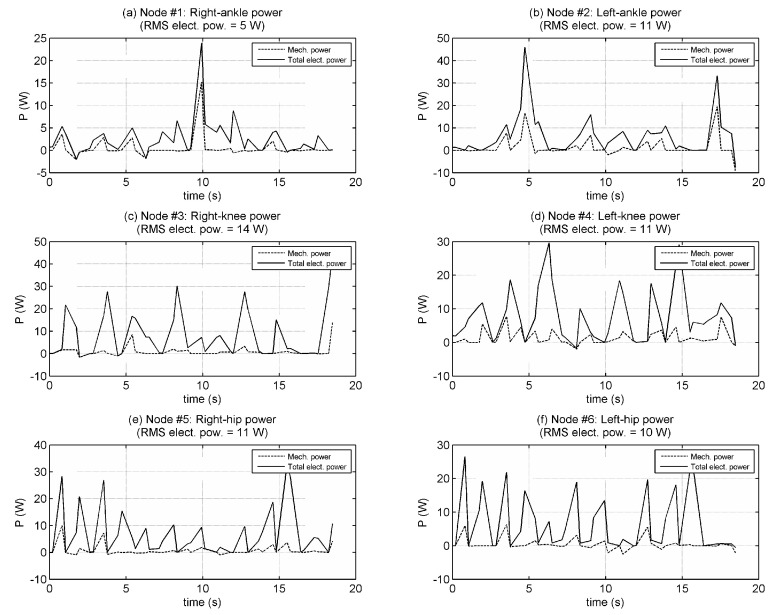
Mechanical power output and total electrical power input during walking test.

Figure 12 shows power measurements while walking. Total electrical power is the sum of mechanical power output and Joule power loss. The RMS powers (for left-right average) are measured as 8 W for ankle joints, about 12.5 W for knee joints, and 10.5 W for hip joints, which totals 62 W RMS for the whole robot.

**Figure 13:**
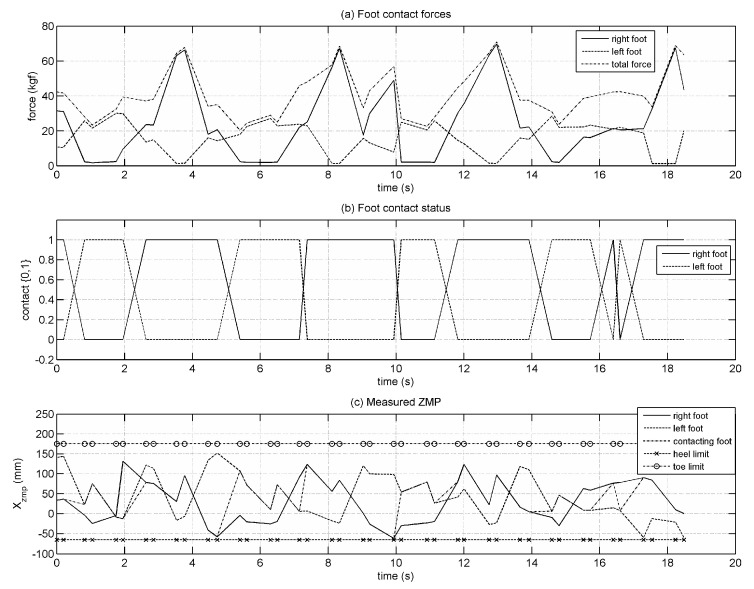
(**a**) Ground reaction force; (**b**) Ground contact; (**c**) Measured ZMP.

Foot–ground contact information was collected by FSR-type force sensors. These sensors could provide qualitative data on contact conditions rather than measuring contact forces accurately. Force measurement using these sensors leads to large errors. Figure 13 shows the total force measurement, contact status and the ZMP criterion for each foot. Results show that the robot can take four stable steps. The ZMP criterion is a measure of the robot’s stability during walking. The ZMP location reached at most 3 mm to heel limit, 51 mm to toe limit and stayed an average distance of 137 mm to toe limit. These results indicate that the ZMP location stays within the support region of the standing foot and confirm that the robot could achieve stable walking without tipping over.

Walking speed, vertical speed and orientation of the torso were computed during walking. The horizontal speed of progression reached a maximum of ~200 mm/s, while its average was around 36 mm/s. Minor oscillations in vertical velocity with an RMS value of 47 mm/s were observed.

## 4. Discussion

The results obtained from the YU-Bibot robot platform provide valuable insights into its walking performance, stability, and potential use cases. The mechatronic design approach of the YU-Bibot platform, influenced by principles of biomimetics, is a strength. The integration of mechanical, electrical, and control components was carefully considered, resulting in a robust robot design. Drawing inspiration from biological systems allows for improved performance and adaptability in real-world environments. For instance, the YU-Bibot platform includes compliant knee and ankle joints. Compliant joints counteract the robot’s weight and compensate for backlash, resulting in improved stability and adaptability to uneven terrains [24]. The results demonstrate that the YU-Bibot robot platform can achieve stable walking. The ZMP criterion revealed that the robot maintained its stability, with the ZMP location remaining within the support region of the standing foot [21]. This highlights the robot’s ability to maintain balance and avoid tipping over during locomotion. Moreover, the robot exhibits accurate tracking of the height reference trajectory during the height posture tests. The small variation in the horizontal and vertical torso positions indicates effective control of the robot’s height [25]. This capability is crucial for adapting to different terrain and maintaining stability in various walking scenarios.

The YU-Bibot platform holds great potential as a research tool for studying bipedal locomotion and mechatronic design. Its biomimetics-inspired approach can provide insights into developing more agile and adaptable robots for various applications. For instance, the platform’s walking and height posture control capabilities make it suitable for applications in humanoid robotics. It can be utilized for human-like locomotion in scenarios that require bipedal robots to navigate complex environments, such as disaster response or service robotics [26]. The YU-Bibot platform could find applications in the field of rehabilitation and assistive devices. After refining of the control algorithms and integration of additional sensing capabilities, it could serve as a walking aid for individuals with mobility impairments, helping them regain independence and improve their quality of life [27]. The YU-Bibot robot platform offers an excellent educational resource for teaching robotics, mechatronics, and control systems. Its modular design and biomimetics principles provide a practical learning experience for students and researchers interested in the field of robotics [28].

This study has a number of limitations. The walking tests demonstrated the YU-Bibot robot’s ability to bear its weight and achieve stable walking. The generated walking gait, represented by the joint motion trajectories, showcased the coordinated movements necessary for locomotion. The power measurements during walking indicated moderate power consumption, with the ankle and hip joints exhibiting lower RMS power compared to the knee joints. This suggests that optimization efforts could be directed toward reducing power consumption at the knee joints. Minor oscillations in vertical velocity were observed during walking. Although the average vertical speed was reasonable, reducing these oscillations would improve stability and enhance the robot’s performance. Additional investigation into the causes of these oscillations and potential control strategies could be beneficial. Nonetheless, the results show the potential of the developed robot for various applications.

## 5. Conclusions

In this study, a design and prototyping approach for planar biped robots were presented and a resulting robot prototype was developed. The design method was primarily based on simulations of natural human gait. The prototype of the robot used foot contact sensors to detect ground reactions and an inertial measurement unit to collect information regarding its orientation and angular speeds. Therefore, low-cost FSR-type sensors can be utilized for ZMP measurement and control. It was observed that the robot can follow static posture commands quite accurately. Results indicate that using a biomimetic optimization-based mechanical design approach can be an alternative to conventional design methods. The developed platform can serve as a test bed for future research on biped robotics with an emphasis on online sensor adaptations and dynamic gaits.

## Figures and Tables

**Table 1:** Masses and dimensions of primary robot parts.

Section	Mass (kg)	Section	Length (mm)
Torso	9.6	Ankle to heel	80
Thigh	2.7	Ankle to toe	160
Shank	3.2	Ankle to sole	34
Foot	1.1	Shank, thigh	460

**Table 2:** Stance leg joint power and torque values (RMS) for one step with several heights and materials.

Sample	Material	Height (m)	Hip Joint		Knee Joint		Ankle Joint	
			Moment (Nm)	Power (W)	Moment (Nm)	Power (W)	Moment (Nm)	Power (W)
1	Steel	1.50	33.5	32.2	87.1	105.4	163.9	145.4
2	Steel	1.75	50.5	47.8	156.2	191.2	292.6	260.5
3	Steel	2.00	75.0	69.6	260.8	321.5	485.2	433.6
4	Aluminum	1.50	11.7	11.3	30.6	37.0	57.6	51.1
5	Aluminum	1.75	17.7	16.8	54.9	67.2	102.7	91.5
6	Aluminum	2.00	26.3	24.4	91.6	112.9	170.4	152.3
7	Aluminum	1.75	10.1	8.5	44.8	73.2	86.4	80.1
8	Carbon fiber	1.75	5.0	4.2	22.2	36.4	42.9	39.8

## Data Availability

The data presented in this study are available on request from the corresponding author.
